# The International ‘Balint’ Award – a rising opportunity for Romanian Medical Students

**Published:** 2010-02-25

**Authors:** A Lală, G Bobîrnac, R Tipa

**Affiliations:** ‘Carol Davila’ University of Medicine and PharmacyRomania

**Keywords:** psychotherapy, Balint method, , Ascona model case presentation

## Abstract

The International ‘Balint’ Award for students, instituted by the Foundation for Psychosomatic and Social Medicine in honor of Michael and Enid Balint, has been a rising opportunity for Romanian medical and psychology students to achieve international fame.  Romanian students have been among the winners of this award for the last 10 years, in competition with students from Ivy League and other illustrious universities.

The ‘Ascona model’ case presentation debates the psychological side of a medical case, while keeping in focus the diagnostic, pathology and treatment issues. This article focuses on explaining this type of case presentation in correlation with one of the papers submitted in the contest that has received this award in the 15^th^ International Balint Congress.

The exposed case is that of a 17–year–old boy presenting with apparent stupor encountered by an emergency mobile unit. The patient was suspected of substance abuse and overdose but these suspicions were denied by the clinical exam. Further encounters led to the conclusion that both the boy and his whole family needed psychotherapy counseling and were referred there with great success.

## Introduction

Michael Balint is a well–known psychologist, born in Budapest in 1896 and the pioneer of what is now known as ‘the Balint method’ case presentation or ‘Balint groups’ for general practitioners around the world.  His work has inspired current group–therapy methods in an important manner and his ideology in psychotherapy and doctor–patient relationship setting is carried on by the International ‘Balint’ Foundation (IBF), established in 25 countries around the world. 

The Ascona Prize for students was founded in 1976 in the honor of Michael and Enid Balint. This prize means to encourage medical and psychology students to develop moral and affective introspective and analytical techniques while presenting an experience they had with a person in suffering, either a patient or an ill family member. The focus is on exposing the student's personal reflections and feelings in response to the psychological solicitation of the experience, of relating with a patient who is revealing not only his medical issues, but also his social, psychological or existential crisis. The main request is that of a scientifically relevant exposition with great emphasis on the student/extern's own impressions and emotions from his point of view – that of an intermediary between parts, a ligand in the actual doctor–patient relationship, having the goal of both to matter in the improvement of that relationship and to pursue his own educational process.

The tools of the trade for this contest are originality, good documentation of the patient's psychological and physiopathological condition and a sincere, analytical, introspective and straightforward presentation.  Emphasizing on the patient's psychological pattern, the student's difficulties in communicating and relating, the analysis of the interactions that occur in the triangle formed by the patient, the student and the attending physician is of great value, this differing from a usual medical or psychological case presentation. The goal, for the student, is to exemplify and reveal conclusions relevant for an improvement in these relationships, and, thinking in perspective, to improve his future feedback and feed–forward patterns. Also aiming at a ‘slight but important change in the personality of the doctor’, as M. Balint described the result of his groups, the involvement of students in this kind of work may be the fundament of a new and better paradigm in medicine, one that will take into greater consideration the psychological aspects of a patient's suffering and illness. 

This has been a great opportunity for students to make their selves known and to achieve international fame. For the last 10 years, Romanian medical students have constantly been among the laureates and have presented their papers in international congresses worldwide. In competition with students from Harvard Medical School, London University or Freiburg Universität, medical students from ‘Carol Davila’ University of Medicine and Pharmacy have won several awards and have been invited year by year  to market both their name and the name of their university. The current, ongoing edition has a paper submission deadline for December 31, 2010, and details can be found on the International Balint Foundation's website.

## Ascona model case presentation

According to IBF, papers are judged according to four criteria, as listed below. For each criterion, there is an example, abstracted from a reference work that has received the Ascona Prize at the 15^th^ International Balint Congress, held in Lisbon. The length of the paper ranges from 3000 to 1000 words, but a more extended analysis is required in order to fulfill all the criteria.

1. Exposition: The paper should include a presentation of a truly personal experience of a student–patient relationship.
  *Being a first year medical student does not give you a large opportunity to interact with patients. Having that in mind and trying to find a way to correct it, I've enrolled in the Emergency and Resuscitation Medical Ambulance system as a volunteer, hoping this will help me make a good idea of what medical practice is all about, and also create a vision of what my future patients could be going through before arriving at the hospital. After receiving the Basic Life Support diploma I had the chance to work with some very good doctors and take part in the solving of medical cases of all sort, many of them being experiences from which I have learned things that will probably guide me through my future career in taking the people's physical and psychic health.
*
  During a night route, at 1:20 AM we received the chart of a 17–year–old boy suspected of a drug overdose. I have been to overdose cases before, but the doctor I was working with that night, went over the things we should be doing upon arrival at the patient's house. The age of the patient and the fact that his father was the one who called the ambulance was at first thought of as a strange thing, most of our overdose patients being over 20 years old and in a distinctly bad social situation.
  Arriving at the house – a 2 room flat at the 2nd floor of a fairly good neighborhood, we were greeted by the patient's extremely frightened mother that quickly guided us to one of the rooms where the 17–year–old was sitting on a chair facing the wall in front of him, glaring at it with an absent, almost ghostly look. I have to say that I was more impressed by this child's look than I was of the patients in much more horrible situations, thinking about it later. I arrived at the conclusion that it was because of his age and the fact that, as I was to find out, he was very scared and hopeless. It was my first–encounter with a seemingly conscious patient, who was not responding to any questions or other means of interaction.
2. Reflection: A description of how the student experienced this relationship, either individually or as part of the medical team.
  Finding myself alone with the patient in the other room, I noticed that he finally started to have some normal behavior such as looking around, blinking, he even looked straight at me. Meeting his sight, I took the chance to connect with him and asked him questions that looked more like questions a friend would ask, rather than a doctor. I asked him why he thought his parents suspected him of drug consumption and I was very surprised by the fact that the young man who 5 minutes earlier was just sitting in a chair looking point–blank at the wall in front of him, not even blinking, was talking to me as any normal person would. He told me that ‘this’ was going on for over 6 months, that his father was constantly ‘harassing’ him and that he just would not accept any arguments that he brought in his defense, therefore he had chosen to completely ignore him and everybody else in his family.
  I asked him about his relationship with his sister and he told me that, because of the fact that his father was forcing her to spy on him, he had to ‘give her up too’, adding that she has ‘her own problems to take care of’, since she was in her graduate year and had to prepare for her exams. He also told me that the only person that he is sorry for is his mother, but that he could not get close to her either because she was under the father's strict authority. It was very hard for me to ask him whether he was actually taking drugs or not, but when I did, I got an answer which I probably should have expected: receiving my question with a smile he told me ‘it would have probably been better’ if he did, but he does not. I asked him what he meant by that and he told me that he could never take drugs, especially paint thinner – what he was accused of, because he knows ‘what those things can do to you’, and, in a burst of laughter he said that even if he wanted to, he didn't have the money to do it anyway.
  Then I asked him if he had any explanation for the smell that seemed to be the root of all these problems and he told me that he had started smoking almost a year before and had arguments about that, afterwards his father had started accusing him of more serious things that he ‘honestly did not do’. He even started to confess that he ran away from home for 2 days a couple of months before because of the strict severity that he ‘was forced to endure’ there. When I asked him about his school results, he looked satisfied and said that was not one of his problems, that he is trying to keep up under the circumstances and that he cannot wait to finish high school and gain his independence. Realizing that I had made some progress in communicating with him, I tried to be sensitive to all his needs in order to maintain his morale, but it was hard for me at this point to help suggest any decision or strong advice because the situation was very delicate and complicated. It seemed rather weird to me that he would open up so quickly and sincerely, I had no explanation for that but I was glad it was happening because comparing his situation when we entered the house with the one he was experiencing then, there was an obvious evolution for the better.
3. Action: The student's own perception of the demands to which he or she felt exposed and an illustration of how she/he responded.
  At that point, I was very confused; I could not understand how a person could go so quickly from a state in which we suspected him of schizophrenia to one that was fairly normal for a child of his age. I asked him if he ever tried to cooperate with his father in finding a solution to their arguments but he replied that it doesn't matter, that he plans to leave this home for good the moment he turns 18; also after telling me this he smiled and said that he couldn't wait for that moment to come. My confusion got even more intense when I realized that I was about to fall into the trap of considering that there was nothing wrong with the person in front of me, and that I was beginning to forget my role there.
  During the conversation I often thought that I was very lucky not to have the problems I was being told of, which made me even more reluctant to try to give him any advice, because I was considering myself not to be the right person to do so. Although being consumed by them, it was not very hard to hide those feelings from the patient
  Returning to the first room to talk to the doctor I found that she was expecting me and looked like she had something important to say to me. She told me that she had talked to the father and that the biggest problem was there, not with the son, responded by telling her what I had found out in the other room. It seems that the father and son had not spoken a word to one another in 6 months and that the situation was critical. The doctor explained to the father that there was no problem with his son and that she was almost sure that he did not even consume drugs, let alone the fact that he was an addict or suffered an overdose. At this point, the father hit the ground with his foot saying, ‘no, I know better, he is an addict and I want you to help me put him into a rehabilitation center’. Knowing that I had an experience with patients that had undergone drug rehab and hoping that there was a small chance that the father would respond to that, the doctor asked me to explain to him what treatment in that sort of place implies, as she went to give the child a final exam.
  I began by assuring the father that I had a good experience in working with addicts and rehab centers, because I was part of an association that activated in the field of post–addiction maintenance of ex drug consumers, but I didn't want to confront him directly with my conclusion that his son was not one of those cases. Therefore, I started to tell him stories that I thought would get his attention and maybe change his perception. I could not get a single doubt in him, that he was wrong. After about 30 minutes of explaining what rehabilitation is all about, all I got from him in response was a ‘well, that was a nice story, kid, but you don't know anything about my problems, that one (referring to his son) needs help and I'm going to make sure he gets it’. Hearing this, I was disappointed and I'm ashamed to say that for a moment I felt like I wanted him to feel for himself what his son was going through. It was the first moment that night that I was scared, I don't know if it was because I didn't know what to do anymore or because I was imagining what the 17–year–old in the other room was about to undergo after we left their house.
4. Progression: A discussion of both ways in which the student's own approach might change in the future, and also possible ways in which future medical training might  enhance the state of awareness for individual students.
  I was and I still am amazed by the complexity of the term ‘medical help’ since, as I found out, you can never draw a line between the physical and the psychical care you have to assure a patient. In order to have a good doctor–patient or student–patient relationship one must firstly take care of the patient's emotional needs or at least try to assure him that they are taken into consideration, otherwise he could be struck by unwanted reactions on the patient's behalf, as I was by the father's indifference to my attempts of broadening his views. The dual aspect of the physician is beginning to be more and more a need than a choice. My student–patient relationship that night was deeply influenced by the excellent communication between the doctor and me. It was very important for me to know that there was an experienced person there that could help me in the difficulties I had in approaching the patients, and I have to say that without the doctor's valuable input, I don't think we could have made any progress in dealing with that family's problems. What I have come to conclude from this aspect will be of great importance in my future decision making concerning the way I communicate with patients.
  First of all, the case I have presented is not a common one and unlikely to come about in the practice of the majority of medical students, but I have to emphasize the fact that, because of its strong empathic requests, it is a marker for the importance of the doctor/student's desire and the capability to acknowledge and understand every aspect of his patient's sufferings, either pathological or not, before proceeding to offer the actual medical care.
  Moreover, it should be taken into account that some situations that on first view seem not to have anything in common with the usual medical act, should be approached with great care, because, if untreated, they may derive into even more complicated one. This widens the area of the level of competence of any doctor or student who is working under the pressure of the emergency medical act.
  In addition, the involvement of medical students in the earlier mentioned cases should be closely observed by a competent doctor and should invariably be doubled by a close doctor-student collaboration, which comes not only in the student’s aid but also in the patient’s. This is especially true for situations like the one I presented when the time of interaction with the patient is short. Showing a good relationship and collaboration between parts should be considered of vital importance by any medical team in the act of treating a patient.
  In conclusion, I  must mention the importance of the fact that any medical student or doctor is aware of the possible conditions that have driven a person to show up as a patient and the way those conditions are acting as factors of influence in the patient's receptiveness to advise, treatment compliance and feedback. The therapist must be aware of every aspect of a patient's emotional state; this, in some cases, implies the co–working of a part that has the best chance of receiving the necessary information and a part that is specialized in treating the illness or preventing it. The refusal to work in these conditions due to possessiveness over an area of expertise or lack of confidence between parts should be avoided if taking into consideration the patient's wellbeing.

## Conclusions

As a medical student, there are many chances to make yourself noted and to set your vision of a situation, make your scientific beliefs known and market your name and your university, which, in return, has great advantages from both the clinic and academic point of view. Congresses, conferences, symposiums and competitions like the one presented above are very good ways to interact with the scientific world and complete the common information supplied at every university with new, debatable and researchable subjects.  Opportunities like the Ascona prize for Students do not receive the publicity that they very well deserve, but this should not be a problem if medical students were to relate more with the scientific world either by participating at events or by registering in academic societies according to their goals and interests.

Gathering and filtering information, consulting medical journals and having a feedback for the medical community is the best approach to the science for every medical student, since this is a domain with an incredible dynamics, and one cannot stand still while the world is revolving around him.

